# PM-MCD: A network combining pyramid feature extraction and multi-scale attention fusion for multiclass change detection

**DOI:** 10.1016/j.isci.2026.114897

**Published:** 2026-02-03

**Authors:** Yingjie Fan, Xiaobing Yang, Boxu Li

**Affiliations:** 1 School of Information Engineering, China Jiliang University, Hangzhou 310018, China

**Keywords:** Remote sensing, Computer modeling

## Abstract

Multiclass change detection in remote sensing images plays a vital role in remote sensing applications. However, the existing methods still have the problem of subtle changes missed. In this paper, we propose a model named PM-MCD, which consists of a VMamba-based pyramid feature extraction encoder for remote sensing images and a multi-scale information aggregation decoder based on MLP and MSSC module, enabling efficient multiclass change detection in remote sensing images. In addition, we propose a multi-scale attention fusion module, MSSC, to enhance the model’s ability to recognize small-scale change regions. Experimental results show that, on the WHU-CD, Landsat-SCD, and CNAM-CD datasets, our model outperforms existing CNN- and Transformer-based methods, achieving 99.4/96.77/90.86% overall accuracy (OA), 90.18/82.27/68.50% mean intersection over union (mIoU), and 91.44/89.88/79.86% F1 scores.

## Highlights

• An end-to-end multiclass change detection model for remote sensing images
• Multi-scale and attention mechanism are used to enhance the model’s performance
• Achieve superior performance on multiple datasets

## Graphical abstract

**Figure:**
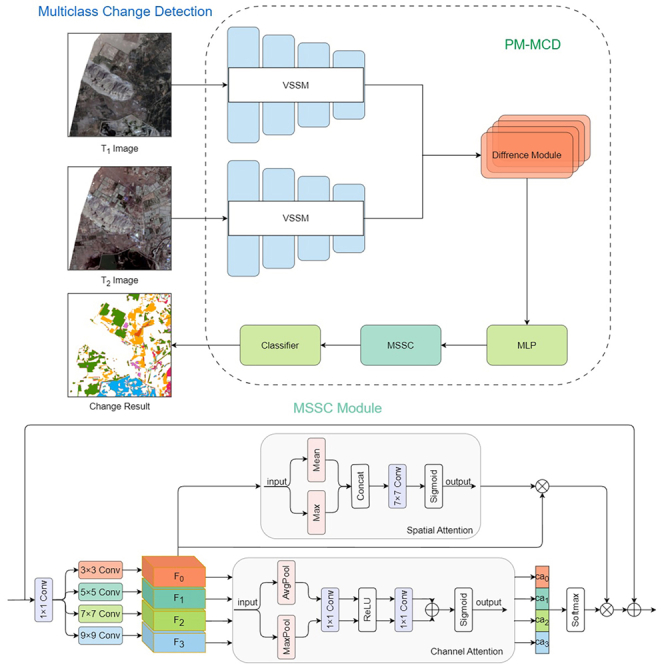

## Introduction

Remote sensing image change detection is an important research direction in the field of artificial intelligence. It focuses on how to effectively detect and classify various changes caused by natural processes or human activities from remote sensing images acquired at different times.^1^^,^^2^ After decades of development, change detection has been widely applied in urban planning, environmental monitoring, disaster management, and other domains.^3^^,^^4^^,^^5^ Furthermore, with the continuous advancement of remote sensing technology, large volumes of high-resolution remote sensing images can now be rapidly obtained. These images provide rich texture and structural information and meet the requirements of various tasks that demand high spatial resolution.^6^^,^^7^^,^^8^ For example, in small-object detection tasks, researchers have proposed adaptive downsampling and scale enhanced detection head to improve the recognition of fine-grained targets.^9^ These efforts further highlight the importance of handling scale variations and preserving detailed information in high-resolution imagery.

**Figure 1:**
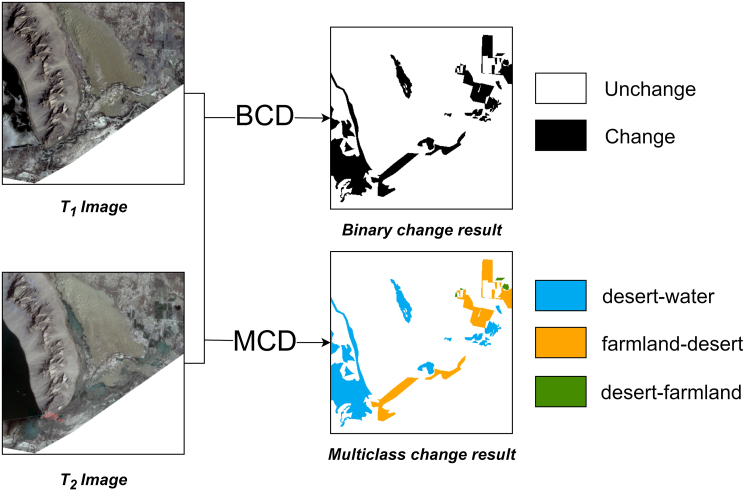
Illustration of the BCD and MCD tasks

Most existing change detection methods primarily focus on binary change detection (BCD), which distinguishes only between changed and unchanged regions.^10^^,^^11^^,^^12^ However, this does not meet the requirements of more complex tasks today. In contrast, multiclass change detection (MCD) can identify multiple types of changes—for example, urban expansion, vegetation cover alteration, and waterbody transitions—thereby offering more detailed information to support the understanding of surface evolution.^13^ An illustration of the BCD and MCD tasks is presented in Figure 1. However, because MCD requires semantic-level discrimination, it poses greater challenges in feature fusion, class imbalance handling, and cross-scale representation learning. Similarly, the field of video processing faces modeling difficulties such as multi-scale redundancy and spatiotemporal consistency. To address these issues, researchers have proposed approaches such as structural simplification and salient-region enhancement (e.g., SETR-Net,^14^ SS-Net^15^) to reduce redundancy and strengthen the modeling of important regions. These ideas offer meaningful inspiration for optimizing feature representations in MCD tasks.

Over the past decade, deep learning-based methods have been widely applied in the field of change detection, effectively identifying the temporal changes of surface objects. Convolutional neural networks (CNNs) have been widely applied to change detection tasks due to their local receptive fields, weight sharing, and pooling operations, which enable efficient extraction of local image features.^16^^,^^17^^,^^18^ For example, FC-Siam-Conc and FC-Siam-Diff^19^ are fully convolutional Siamese networks equipped with skip-connection structures. FC-Siam-Conc concatenates feature maps to preserve rich information, whereas FC-Siam-Diff computes feature differences to more directly focus on regions of change. MFCN^20^ extracts features of ground objects at multiple scales through multiscale convolution kernels and employs weighted binary cross-entropy together with a dice coefficient loss to address unbalanced samples, thereby improving the accuracy of change detection in high-resolution remote sensing imagery. However, CNNs primarily capture local features and struggle to model long-range dependencies effectively, which often leads to missed detections of small-scale changes or blurred boundaries.

To compensate for the limited global modeling capability of CNNs, Mei et al.^21^ designed a depth feature interaction (DFI) module, which injects CNN-derived local features into MobileSAM, enabling the model to jointly capture global and local contextual information. Chen et al.^22^ employed large-kernel convolutions and multi-attention mechanisms to balance global semantic modeling with local detail detection, thereby improving change detection accuracy in high-resolution remote sensing imagery. In addition, Transformer has gained considerable attention due to its superior ability to capture global dependencies. Transformer and its variants^23^^,^^24^^,^^25^ have been widely introduced into change detection tasks. For example, HATNet^26^ incorporates a hybrid attention mechanism and performs multi-scale feature alignment within a transformer framework, significantly enhancing both accuracy and robustness in building change detection. Models such as ChangeMask^27^ and ChangeFormer^28^ also demonstrate strong global modeling abilities, yet they suffer from high computational cost and substantial memory consumption inherent to self-attention mechanisms. Similar approaches have been employed in other computer vision tasks to capture global contextual information. For instance, SDFF-Net^29^ leverages a layer-by-layer correlation aggregation (LCA) module to integrate semantic and detailed features in a top-down manner, enabling the model to attend to both local details and global context. PMVOS^30^ combines a pyramid multi-scale architecture with the long-short-term transformer modules of PLSTT to establish global dependencies across scales and temporal keyframes, thereby effectively extracting global contextual information.

Although transformer can capture global contextual information through its self-attention mechanism, their computational complexity scales quadratically with sequence length. The recently proposed Mamba^31^ model introduces a selective scan mechanism and leverages a state space model (SSM) to achieve linear-time global modeling, demonstrating strong performance in sequential tasks. VMamba^32^ extends Mamba to the visual domain by adopting a 2D selective scan (SS2D), which effectively captures global context while significantly reducing computational overhead. In the field of remote sensing change detection, researchers have already applied the Mamba architecture to high-resolution image analysis.^33^^,^^34^ For example, Chen et al.^35^ were the first to introduce the Mamba architecture into remote sensing change detection, employing VMamba as an encoder to comprehensively learn global spatial contextual information from input images. They further designed three spatiotemporal relation modeling strategies—spatiotemporal sequential, spatiotemporal cross, and spatiotemporal parallel—within the change decoder to fully exploit spatiotemporal information. Huang et al.^36^ proposed LCCDMamba, a land-cover change detection network based on visual state space models. It employs a Siam-VMamba backbone to extract multi-dimensional land-cover features and integrates local and global information through a multiscale information spatio-temporal fusion (MISF) module. Additionally, they design a dual token modeling SSM (DTMS), which enhances the model’s ability to process cross-dimensional change features, thereby mitigating information loss. These studies demonstrate that the Mamba architecture achieves strong global modeling capability while maintaining computational efficiency, offering new insights for addressing spatiotemporal feature consistency and multi-scale change recognition in MCD.

In high-resolution remote sensing imagery, small-scale targets often exhibit tiny sizes, irregular shapes, and weak textures. During multi-stage feature extraction, deep models tend to smooth or suppress these subtle details. Furthermore, the processes of feature downsampling and scale fusion may blur true change boundaries, making it difficult for the model to accurately localize edge regions. Consequently, this leads to missed detections of small-scale changes and discontinuous boundaries, especially in densely structured or complex background areas. Such limitations directly affect the precision and reliability of change region detection. Therefore, it is essential to design models that can preserve fine-grained spatial information and enhance feature representation across scales to effectively capture small-scale changes. This paper proposes a novel MCD framework for remote sensing images, named PM-MCD. PM-MCD effectively leverages global contextual and multi-scale information from multi-temporal images. First, the encoder is based on the VMamba architecture, which not only inherits its ability to model long sequences but also further exploits its advantage in global contextual awareness, thereby enabling more robust localization of subtle changes in complex backgrounds. Next, during the decoding phase, PM-MCD introduces a decoder structure consisting of MLP layers and an MSSC module. The MSSC module enhances cross-scale feature fusion by combining multi-scale feature extraction with channel and spatial attention mechanisms, addressing the limitations of existing Mamba-based methods in multi-scale modeling. With these improvements, PM-MCD can more accurately capture changes in the image while maintaining robust detection of subtle changes even under significant background interference.

The main contributions associated with the development of the PM-MCD are as follows.

(1) Based on the VMamba architecture, an end-to-end MCD network, PM-MCD, is proposed, providing an efficient and feasible approach for MCD tasks.
(2) We propose a module named MSSC, which can effectively extract small-scale change information from the feature map. The MSSC module is a flexible and extensible module that can be used to improve performance in other networks. Additionally, we conducted extensive ablation experiments to demonstrate the effectiveness of MSSC.
(3) Extensive experiments were conducted on the publicly available remote sensing dataset WHU-CD, Landsat-SCD, and CNAM-CD, demonstrating the superiority of the PM-MCD.

## Results

**Figure 2:**
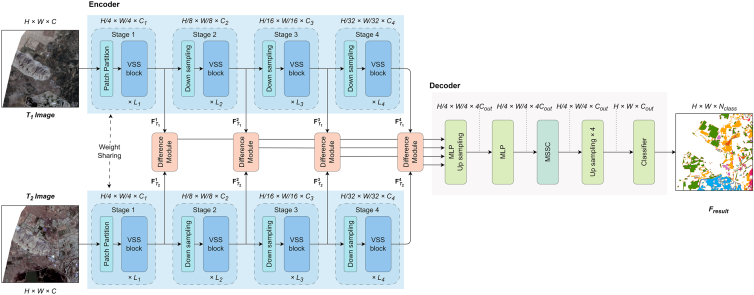
Overall architecture of the proposed PM-MCD

This paper designs a network combining pyramid feature extraction and multi-scale attention fusion named PM-MCD (Figure 2). The proposed model consists of three components: a VMamba-based siamese encoder for extracting global features from multi-temporal images, four difference feature extraction modules for capturing difference features at multiple levels, and a multi-scale attention decoder for fusing these multi-level difference features and predicting MCD information. Our model enhances feature representation by incorporating VMamba and the proposed MSSC module. Specifically, the Siamese encoder leverages VMamba to better capture the global spatial-temporal context of multi-temporal images, thereby improving the generalization capability of the model. In the decoder, the MSSC module is designed to integrate multi-scale features, which enables the effective capture of fine-grained details in small-scale changes.

### Comparative experiment

To demonstrate the superiority of the PM-MCD based on the VMamba architecture in CD tasks, we conducted comparative experiments with several advanced methods based on CNN and Transformer architectures. The effectiveness of the proposed method was validated on the WHU-CD,^37^ Landsat-SCD,^38^ and CNAM-CD^39^ datasets. All models were evaluated under the same experimental platform, with training parameters kept consistent with the original papers.

The detection results for the WHU-CD dataset are detailed in Table 1, where a quantitative comparison of the methods is provided. Although other methods perform well, our method still stands out. It outperforms the second-best method, NATCD, by 1.12%, 2.42%, 0.2%, 2.2%, and 2.06% in terms of Pre, Re, OA, mIoU, and F1, respectively. These improvements highlight the superiority of our PM-MCD.

**Table 1:** The overall quantitative results of different methods on the WHU-CD dataset

Type	Method	Pre	Rec	OA	mIoU	F1
CNN	SGSLN^40^	86.19	85.70	98.77	83.94	85.30
	CACD2Net^41^	86.12	89.16	98.86	85.00	86.97
	MetaCD^42^	87.12	86.00	98.95	84.03	85.66
	Siamese Attention U-Net^43^	74.46	72.99	97.94	71.14	72.88
Transformer	TransUNetCD^44^	87.08	84.29	98.70	83.10	84.69
	NATCD^45^	91.84	88.84	99.20	87.98	89.38
	Pyramid-SCDFormer^38^	85.08	82.87	98.60	81.03	82.87
Mamba	PM-MCD(Ours)	92.96	91.26	99.40	90.18	91.44

**Figure 3:**
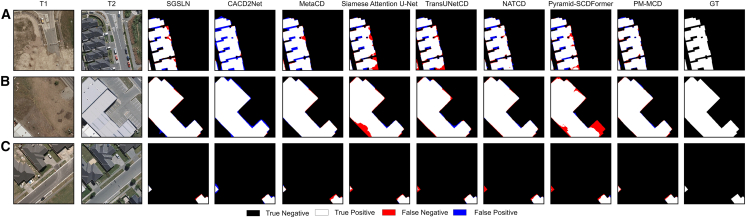
Visualization results of different methods on the WHU-CD dataset (A–C) Visualization results of three pairs of images with different models on the WHU-CD dataset.

Figure 3 Visualization results of different methods on the WHU-CD dataset presents a visual comparison of different methods on the WHU-CD dataset. To enhance visual clarity, we use different colors to highlight key and detailed results, with red and blue representing missed detection and false positives, respectively.

From Figure 3A, it can be seen that when the shape of the building is relatively complex, our method can clearly identify the shape of the building, while other methods show large areas of false positives in the gaps between buildings. Additionally, methods such as Siamese attention U-Net and TransUNetCD show obvious missed detection issues. In the large change regions (Figure 3B), Siamese attention U-Net and pyramid-SCDFormer suffer from severe missed detections. In the small change regions (Figure 3C), TransUNetCD, NATCD, and pyramid-SCDFormer all have large areas of missed detection. In contrast, our method shows superior visual performance, excelling in boundary handling and small object detection.

Table 2 lists the quantitative comparison results of different methods on the landsat-SCD dataset. As can be seen from the table, our method achieves 88.52/96.77/82.27/89.88% in Rec, OA, mIoU, and F1, respectively. Compared to the second-ranked Siamese attention U-Net, our method improves Rec, OA, mIoU, and F1 by 4.31%, 1.44%, 3.14%, and 1.91%, respectively. Although Siamese attention U-Net achieves the highest pre value, our method significantly outperforms other methods in the remaining four metrics, further proving the feasibility and superiority of our method in the MCD.

**Table 2:** The overall quantitative results of different methods on the landsat-SCD dataset

Type	Method	Pre	Rec	OA	mIoU	F1
CNN	SGSLN	72.12	59.22	89.31	49.76	63.61
	MetaCD	87.82	83.66	95.40	75.61	85.54
	Siamese Attention U-Net	92.61	84.21	95.33	79.13	87.97
Transformer	TransUNetCD	86.58	82.77	95.46	74.31	84.57
	NATCD	82.78	73.82	93.34	65.38	77.78
	Pyramid-SCDFormer	88.02	81.87	95.20	74.33	84.63
Mamba	PM-MCD(Ours)	91.39	88.52	96.77	82.27	89.88

**Figure 4:**
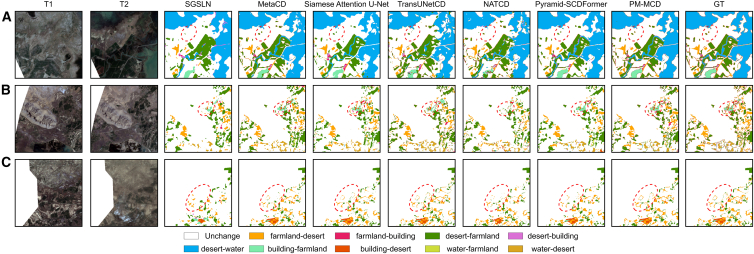
Visualization results of different methods on the landsat-SCD dataset (A–C) Visualization results of three pairs of images with different models on the Landsat-SCD dataset.

Figure 4 shows the performance of different CD methods on the landsat-SCD dataset, providing a clear visual comparison of the performance of each method.

In areas with small-scale changes (as shown in the red circles in Figure 4A), MetaCD, Siamese attention U-Net, pyramid-SCDFormer, and our method can accurately identify the occurrence of subtle changes, but our method is visually the most similar to the ground truth.

In areas with dense changes, the data may contain multiple change categories, such as building demolition, new construction, and land cover changes. The red circle in Figure 4B shows that our method significantly outperforms other methods in terms of recognition accuracy in change-dense areas, with a better ability to distinguish between different types of changes. Other models tend to produce false positives or missed detections in such scenarios. For example, pyramid-SCDFormer fails to correctly identify the type of change within the red circle, while NATCD is unable to detect the approximate change occurring inside the red circle.

Detecting small edge changes is crucial in change detection tasks. The red circle in Figure 4C shows that Siamese attention U-Net, TransUNetCD, and our method are able to maintain fine change boundary information, whereas methods like MetaCD have issues such as blurred edges. This indicates that our method effectively reduces boundary errors and improves boundary recognition accuracy.

Table 3 summarizes the network parameters and FLOPs of the compared methods. In PM-MCD-same, the number of layers and channels in each stage is aligned with those of pyramid-SCDFormer. It can be observed that under this configuration, our model yields fewer parameters and FLOPs than several competing models, such as Siamese attention U-Net, TransUNetCD, and pyramid-SCDFormer. Moreover, Table 4 shows that our model also achieves a notable advantage in terms of accuracy. Although PM-MCD itself involves a higher parameter count and computational cost, it delivers corresponding improvements in accuracy.

**Table 3:** Comparison of computational costs of different methods on the landsat-SCD dataset

Type	Method	Params. (M)	FLOPs (G)
CNN	SGSLN	6.05	63.12
	MetaCD	24.93	203.08
	Siamese Attention U-Net	70.40	894.62
Transformer	TransUNetCD	122.20	245.94
	NATCD	101.56	315.80
	Pyramid-SCDFormer	53.51	231.24
Mamba	PM-MCD-same	52.08	231.04
	PM-MCD	114.02	306.90

**Table 4:** The overall quantitative results of different methods on the landsat-SCD dataset

Method	OA	mIoU	F1
TransUNetCD	95.46	74.31	84.57
NATCD	93.34	65.38	77.78
Pyramid-SCDFormer	95.20	74.33	84.63
PM-MCD-same	96.08	78.95	87.72

Table 5 reports the experimental results on the CNAM-CD dataset, from which it is evident that the proposed PM-MCD model achieves the best overall performance among all comparison methods. PM-MCD attains the highest scores across all five evaluation metrics—Pre, Rec, OA, mIoU, and F1—achieving an OA of 90.86%, an F1-score of 79.86%, and an impressive mIoU of 68.50%. These results demonstrate that PM-MCD offers notable advantages in both the accuracy and completeness of change-region detection.

**Table 5:** The overall quantitative results of different methods on the CNAM-CD dataset

Type	Method	Pre	Rec	OA	mIoU	F1
CNN	SGSLN	63.76	63.94	86.18	52.12	63.27
	MetaCD	77.27	72.33	88.40	61.92	74.35
	Siamese Attention U-Net	80.42	74.80	89.59	64.94	77.03
Transformer	TransUNetCD	75.06	65.58	86.90	55.37	67.63
	NATCD	78.87	70.64	88.31	61.22	73.73
	Pyramid-SCDFormer	81.27	70.22	88.95	61.80	73.71
Mamba	PM-MCD(Ours)	84.96	76.91	90.86	68.50	79.86

**Figure 5:**
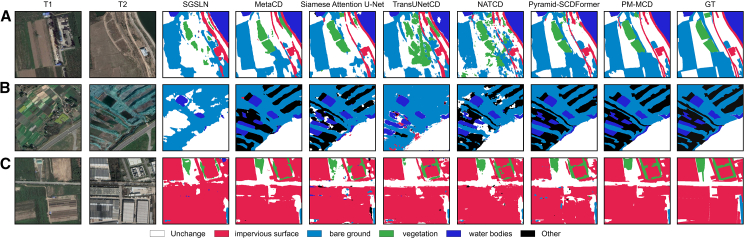
Visualization results of different methods on the CNAM-CD dataset (A–C) Visualization results of three pairs of images with different models on the CNAM-CD dataset.

Figure 5 presents the visual comparison results on the CNAM-CD dataset. As shown in Figure 5A, our method effectively identifies elongated change regions, such as the slender impervious surface area in the example. Although pyramid-SCDFormer is also able to detect this region, its predictions exhibit discontinuities within the same change area. From Figures 5B and 5C, it is evident that our method surpasses others in terms of boundary delineation and the completeness of detected change regions. For example, in Figure 5B, the connected water bodies and other change regions, and in Figure 5C, the connected impervious surface and vegetation classes, are more accurately and coherently captured by our approach, resulting in superior visual outcomes.

**Figure 6:**
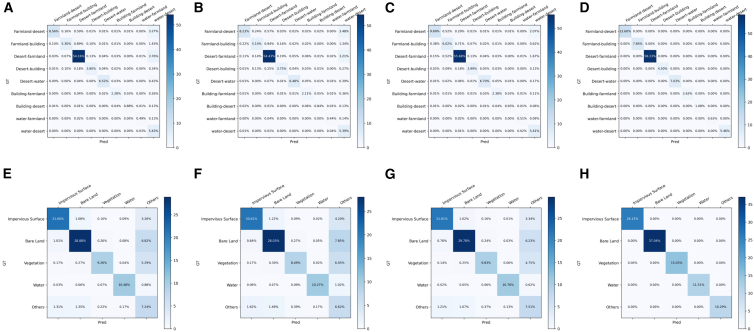
Confusion matrices of different models (A–D) Confusion matrices of Siamese Attention U-Net, Pyramid-SCDFormer, PM-MCD, and the ground truth on the Landsat-SCD dataset. (E–H) Confusion matrices of Siamese Attention U-Net, Pyramid-SCDFormer, PM-MCD, and the ground truth on the CNAM-CD dataset.

We plotted class-wise confusion matrices (excluding the unchanged class) for the landsat-SCD and CNAM-CD datasets, where the horizontal axis denotes the predictions, and the vertical axis denotes the ground truth. Figures 6A–6D illustrate the confusion matrices of Siamese attention U-Net, pyramid-SCDFormer, PM-MCD, and the ground truth on the Landsat-SCD dataset, while Figures 6E–6H present the results of the same models on the CNAM-CD dataset. It can be observed that our method achieves significantly lower false negatives (FN) and false positives (FP) across the major change classes, indicating more stable and reliable class discrimination capability. On the landsat-SCD dataset, our approach substantially reduces misclassifications between key change types such as desert-farmland and farmland-desert, demonstrating enhanced robustness in distinguishing visually similar land-cover transitions. On the CNAM-CD dataset, our method significantly decreases FN in the bare land, vegetation, and water classes, while also reducing FP caused by misclassification from other categories, thereby improving the overall reliability of the major classes.

### Ablation experiments

To validate the effectiveness of the MSSC module, we conduct comprehensive ablation experiments on two datasets. We add the proposed module to networks with different backbone structures to evaluate the performance of our network architecture.

The experimental results detailed in Table 6 illustrate the impact of the MSSC module on network performance on the WHU-CD dataset. MetaCD, NATCD, and our method are experimentally compared. From the results, the model performance has been improved after adding the MSSC module to different networks. Our method improves Pre/Rec/OA/mIoU/F1 by 0.63%, 0.61%, 0.26%, 0.96%, 0.61%, respectively.

**Table 6:** Quantitative ablation experimental results on the WHU-CD dataset

Method	MSSC	Pre	Rec	OA	mIoU	F1
MetaCD	×	87.12	86.00	98.95	84.03	85.66
	✓	89.39	86.78	98.91	86.04	87.53
NATCD	×	91.84	88.84	99.20	87.98	89.38
	✓	92.14	89.90	99.30	88.70	90.05
Ours	×	92.33	89.48	99.32	88.55	90.00
	✓	92.96	91.26	99.40	90.18	91.44

Table 7 shows the mIoU performance of different methods on the landsat-SCD dataset after adding the MSSC module. The experiment compares Siamese attention U-Net, TransUnetCD, and our method. The results show that the MSSC module further improves model performance, particularly for the TransUnetCD model, with a significant improvement. After adding the MSSC module, the mIoU for change categories with less than 1% proportion increased by 5.61%–8.8%, and for other proportional change categories, it increased by 1.59%–7.2%. After adding the MSSC module, our method improved the mIoU for change categories with less than 1% proportion by 0.66%–1.6%, and for other proportional change categories, it improved by 0.33%–1.6%. Therefore, the proposed MSSC module is more effective in improving small proportion change categories.

**Table 7:** Quantitative ablation experimental results on the landsat-SCD dataset

Change type	Change type proportion	Siamese Attention U-Net		TransUNetCD		Ours	
		×	✓	×	✓	×	✓
No change	81.11	94.92	95.09	95.66	97.25	96.73	97.06
Farmland to desert	1.81	62.16	63.14	59.57	66.77	65.26	66.86
Farmland to building	0.95	63.58	64.07	57.15	62.76	62.97	64.03
Desert to farmland	11.61	84.90	85.34	87.15	91.65	89.73	90.68
Desert to building	0.77	79.25	79.89	73.86	80.11	82.09	83.39
Desert to water	2.12	88.11	88.80	87.72	90.19	91.03	91.53
Building to farmland	0.33	80.93	81.49	70.18	78.98	81.92	82.58
Building to desert	0.11	77.13	78.44	65.37	72.35	78.83	79.89
Water to farmland	0.09	71.03	72.42	58.51	66.76	72.05	73.65
Water to desert	1.10	89.29	89.90	87.97	91.76	92.46	93.02

**Figure 7:**
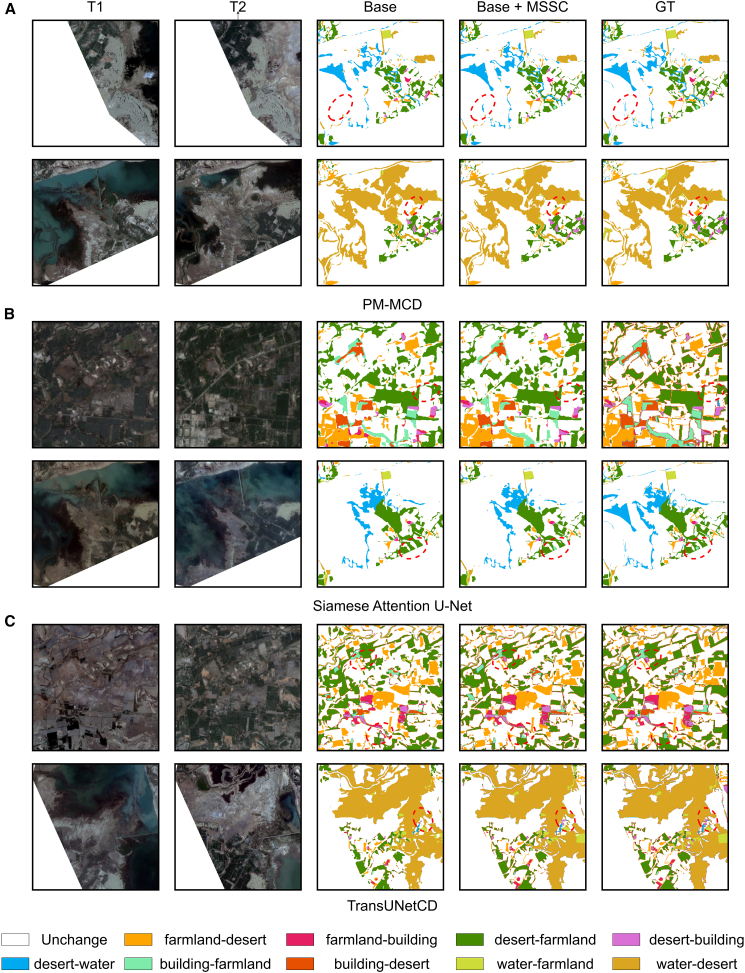
Visualization results of ablation experiments for different methods on the Landsat-SCD dataset (A) Visualization results of ablation experiments of PM-MCD on the Landsat-SCD dataset. (B) Visualization results of ablation experiments of Siamese Attention U-Net on the Landsat-SCD dataset. (C) Visualization results of ablation experiments of TransUNetCD on the Landsat-SCD dataset.

In addition, to intuitively illustrate the role of the MSSC module within the network, we visualized the results of the ablation experiments on the landsat-SCD dataset, as shown in Figure 7. It can be observed that, for our proposed model as well as for Siamese attention U-Net and TransUNetCD, the incorporation of MSSC improves their ability to identify subtle changes. Moreover, for TransUNetCD, the inclusion of MSSC also corrects misclassified change categories.

From the above results, it can be seen that our proposed MSSC module performs excellently on both datasets, proving its effectiveness and superiority in change detection tasks.

Alternatively, we performed ablation experiments on different components in the MSSC module to evaluate the impact of these components individually. As shown by the ablation study results in Table 8, the channel attention (CA) module contributes the most to performance improvement among all submodules. When only CA is added, the Pre, mIoU, and F1 metrics increase by 0.50%, 0.89%, and 0.54%, respectively, representing the highest gains among the individual components. This indicates that CA plays a significant role in modeling feature importance by suppressing redundant responses and enhancing semantically meaningful features. The multi-scale convolution branch (MS) yields the most notable improvement in the Rec metric (+0.54%), demonstrating its crucial ability to capture small-scale changes, which is consistent with the design objectives of the MSSC module. Although the improvement brought by spatial attention (SA) is relatively modest, it still plays a supportive role in enhancing local spatial features. When all three components are combined, the model achieves the best performance across all metrics, indicating that multi-scale feature modeling and dual attention mechanisms are complementary and jointly contribute to the effectiveness of the MSSC module.

**Table 8:** Ablation experiments of different components of the MSSC module

MS	CA	SA	Pre	Rec	OA	mIoU	F1
×	×	×	90.76	87.91	96.51	81.31	89.27
✓	×	×	91.06	88.45	96.65	82.09	89.74
×	✓	×	91.26	88.40	96.68	82.20	89.81
×	×	✓	90.83	88.31	96.66	81.70	89.47
✓	✓	✓	91.39	88.52	96.77	82.27	89.88

MS, CA, and SA, respectively, represent multi-scale convolution branch, channel attention, and spatial attention.

To evaluate the effectiveness of the kernel size design and grouped convolution strategy in the MSSC module, we conducted ablation studies on various combinations of convolution kernels and group settings, as reported in Table 9. The selection of kernel sizes is motivated by the need to capture small-scale changes using smaller kernels, while slightly larger kernels provide richer receptive fields. We do not include larger kernels in the ablation study because excessively large kernels introduce redundant background information, which hinders the detection of small-scale variations, and also significantly increase the model’s parameters and computational cost. Grouped convolution is employed to reduce the number of parameters while enhancing channel independence across convolution kernels, ensuring that each scale-specific branch focuses on its corresponding feature patterns. As shown by the results, when keeping kernel sizes fixed, increasing the group setting from (1,1,1, and 1) to (1,4,8, and 16) improves mIoU and F1 by 0.4% and 0.25%, respectively, confirming the performance gain brought by grouped convolutions. In addition, moderately enlarging the kernel sizes effectively expands the scale coverage. The kernel configuration (3,5,7, and 9) provides broader scale coverage than (1,3,5, and 7), resulting in higher evaluation indicators. Finally, the best performance is achieved with the combination of (3,5,7, and 9) kernels and (1,4,8, and 16) groups, demonstrating that kernel size diversity and group allocation offer complementary benefits.

**Table 9:** The performance of different convolution kernel sizes and group sizes on the Landsat-SCD dataset

Kernel size	Group size	Pre	Rec	OA	mIoU	F1
(3,5,7,9)	(1,1,1,1)	90.84	88.49	96.72	81.87	89.63
(3,5,7,9)	(8,8,8,8)	91.29	88.32	96.69	82.05	89.74
(3,5,7,9)	(4,8,16,32)	91.12	88.58	96.76	82.15	89.81
(1,3,5,7)	(1,4,8,16)	91.26	88.49	96.77	82.17	89.82
(3,5,7,9)	(1,4,8,16)	91.39	88.52	96.77	82.27	89.88

## Discussion

The proposed PM-MCD demonstrates significant performance advantages in multiclass remote sensing change detection tasks, particularly excelling in fine-grained change recognition. Experimental results indicate that the global modeling capability of VMamba, combined with the multi-scale attention fusion mechanism of the MSSC module, effectively enhances the model’s ability to perceive cross-scale change regions, leading to superior performance on multiple public datasets. Nevertheless, despite the strong overall performance of PM-MCD, several aspects warrant further investigation and improvement.

First, although PM-MCD reduces computational overhead compared to Transformer-based methods, it may still encounter challenges related to memory consumption and inference efficiency when processing large-scale, high-resolution remote sensing imagery. Future work may explore more lightweight module designs to enable efficient deployment and practical engineering applications.

Moreover, considering the needs of real-world urban management and ecological monitoring, MCD often requires stronger semantic interpretability and cross-modal information support. Directions such as multi-source data fusion (e.g., LiDAR, SAR) and multimodal learning have great potential to further improve the model’s generalization ability. In future work, we plan to extend PM-MCD to multimodal remote sensing change detection scenarios to enhance its adaptability and practical value in complex real-world environments.

### Conclusions

In this paper, we propose a new network combining pyramid feature extraction and multi-scale attention fusion, named PM-MCD, with the aim of improving the accuracy of MCD on high-resolution remote sensing imagery. By incorporating a linear-complexity global modeling mechanism together with a multi-scale attention fusion module, PM-MCD achieves efficient inference while effectively capturing spatiotemporal change patterns, thereby enhancing its ability to identify small-scale and complex change types. Experimental results demonstrate that PM-MCD achieves superior detection accuracy on datasets including WHU-CD, landsat-SCD, and CNAM-CD.

Furthermore, extensive comparisons and ablation studies verify the effectiveness of the MSSC module in enhancing small-scale feature representation, and analyze how design choices—such as grouped convolution and kernel-size configuration—affect model performance. The results further highlight the crucial role of multi-scale feature fusion and attention mechanisms in MCD.

### Limitations of the study

Nevertheless, PM-MCD still has certain limitations. In the landsat-SCD dataset, building changes account for a relatively small proportion, while the WHU-CD dataset consists solely of building changes but involves only a single change category. The CNAM-CD dataset also has only five categories. To better adapt PM-MCD for urban planning applications, experiments on datasets with a higher proportion of building changes and more diverse change categories are necessary. In future work, we aim to apply the proposed model to urban planning tasks, which require accurate identification of changes related to buildings, vegetation, and other relevant features.

## Resource availability

### Lead contact

Further information and requests for resources and reagents should be directed to and will be fulfilled by the lead contact, Xiaobing Yang (xyang@cjlu.edu.cn).

### Materials availability

This study did not generate new materials.

### Data and code availability

• This paper analyzes existing, publicly available data, the doi is listed in the key resources table.
• This article reports the original code, which has been made publicly available via DOI at https://github.com/fyj0314/PM-MCD.
• Any additional information required to reanalyze the data reported in this article is available from the lead contact upon request.

## Acknowledgments

We are grateful to the editors and anonymous reviewers for their constructive comments and suggestions, which have helped improve earlier drafts of this article.

## Author contributions

Conceptualization, Y.F. and X.Y.; methodology, Y.F. and X.Y.; validation, Y.F.; formal analysis, Y.F.; data curation, Y.F.; writing - original draft preparation, Y.F.; writing - review & editing, Y.F. and X.Y.; visualization, Y.F. and B.L.

## Declaration of interests

The authors declare no competing interests.

## STAR★Methods

### Key resources table

**Table:** 

REAGENT or RESOURCE	SOURCE	IDENTIFIER
**Deposited data**		

WHU-CD	Ji et al.^37^	https://gpcv.whu.edu.cn/data/building_dataset.html
Landsat-SCD	Yuan et al.^38^	https://doi.org/10.6084/m9.figshare.19946135.v1
CNAM-CD	Zhou et al.^39^	https://github.com/Silvestezhou/CNAM-CD

**Software and algorithms**		

Python	Version 3.10	https://www.python.org/
PyTorch	Version 2.1.0	https://pytorch.org/
PM-MCD	Code for this study	https://github.com/fyj0314/PM-MCD

### Experimental model and study participant details

#### Datasets

We conducted experiments on the following datasets.

(1) **WHU-CD Datasets:** This dataset is a collection of high-resolution aerial images of Christchurch, New Zealand, from 2012 to 2016. The spatial resolution of the images is 0.2 meters per pixel. The types of changes mainly include the construction (appearance) and demolition (disappearance) of buildings, reflecting the building dynamics during the post-earthquake reconstruction period. The dataset consists of two large-scale aerial images, each with a size of 15354 × 32507 pixels, covering an area of approximately 20.5 square kilometers. We divide the large images into 256 × 256 pixel patches for use in change detection tasks. We randomly split the dataset into training, validation, and test sets at 8:1:1.
(2) **Landsat-SCD datasets:** The dataset collects Landsat images captured in the Tumxuk region of Xinjiang between 1980 and 2020, providing rich historical data and extensive spatial coverage. The resolution of the images is 30 meters. The dataset provides 10 change types, where each “from-to” change type is a separate category representing land cover transitions. Change pixels account for 18.89% of the total pixels, including transitions such as Building to Farmland, Farmland to Building, and Water to Farmland, and more. The Landsat-SCD dataset consists of 8468 pairs of images, each with a spatial resolution of 416 × 416. The images in the dataset have undergone data augmentation operations, including flipping, masking, and re-scaling. We randomly split the dataset into training, validation, and test sets, with a ratio of 8:1:1.
(3) **CNAM-CD datasets:** The dataset comprises high-resolution Google Earth images collected from 2013 to 2022 across twelve national-level new districts in China. It features extensive spatial coverage and a long temporal span, effectively capturing the real evolution of rapidly urbanizing regions. The dataset categorizes land-cover types into five classes—bare land, vegetation, water, impervious surfaces, and others—and further provides pixel-level multiclass change annotations. CNAM-CD contains 512 × 512 image pairs, totaling 2,503 bi-temporal samples. We randomly split the dataset into training, validation, and test sets at a ratio of 3:1:1.

#### Implementation details

We implemented all experiments on two GeForce RTX 4080 GPU platforms using the PyTorch library. Except for the batch size, all comparative methods were trained using the hyperparameter settings recommended in their original papers. We used the Adamw optimizer^46^ to train the model, with a weight decay of 0.01 and an initial learning rate set to 0.0001.

At the dataset level, WHU-CD is used for binary change detection, with each image of size 256 × 256. Considering the dataset scale and its binary classification nature, we used a batch size of 16 and trained for 100 epochs to balance training stability and computational efficiency. For the multiclass change detection datasets Landsat-SCD and CNAM-CD, the samples are more complex, and the class variations are harder to learn. Thus, we trained for 200 epochs to sufficiently capture multiclass features. In addition, due to computational constraints, the batch sizes were set to 6 and 4, respectively.

Furthermore, for PM-MCD implementation, we used a single-channel binary detection head with binary cross-entropy loss for binary change detection tasks, enabling discrimination between changed and unchanged areas. For multiclass change detection, we employed a multi-channel detection head, where the number of output channels corresponds to the number of change categories. The loss function combines cross-entropy loss with Lovasz-softmax loss^47^ to mitigate the impact of class imbalance. Finally, below table summarizes the final configuration of the proposed model.

**Table:** Configurations of the proposed model

	Layer	Channel	Output size
Stage 1	3	128	104 × 104
Stage 2	3	256	52 × 52
Stage 3	4	512	26 × 26
Stage 4	3	1024	13 × 13

#### Assessment of indicators

To evaluate the performance of our model, five evaluation metrics were used: Overall Accuracy (OA), Precision (Pre), Recall (Rec), F1-score (F1), and mean Intersection over Union (mIoU), which is the average of individual IoU values.

(Equation 1) $$\begin{array}{c} OA=\frac{TP+TN}{TP+TN+FP+FN} \end{array}$$

(Equation 2) $$\begin{array}{c} precision=\frac{TP}{TP+FP} \end{array}$$

(Equation 3) $$\begin{array}{c} recall=\frac{TP}{TP+FN} \end{array}$$

(Equation 4) $$\begin{array}{c} F1=\frac{2\times precision\times recall}{precision+recall} \end{array}$$

(Equation 5) $$\begin{array}{c} MIoU=\frac{TP}{FP+FN+TP} \end{array}$$

where *TP* represents the number of true positive samples correctly classified, *FP* refers to the number of actual negative samples incorrectly classified as positive, *FN* represents the number of actual positive samples incorrectly classified as negative, and *TN* denotes the number of true negative samples correctly classified.

### Method details

#### Siamese encoder

The core of the Mamba model lies in its use of selective scanning operations to focus on relevant parts of the input data while ignoring irrelevant information. This mechanism enables Mamba to maintain linear computational complexity when processing long sequence data, thereby improving its computational efficiency. However, when two-dimensional image data is directly flattened into a one-dimensional sequence for input into Mamba, the model’s expressive power is severely limited due to the lack of spatial contextual information. To address this issue, VMamba extends Mamba and introduces the Visual State Space (VSS) module, specifically designed for visual tasks.

The core of the VSS module (Figure 8) is the SS2D mechanism (Figure 9), which processes two-dimensional visual data through four different scanning directions. These four scanning paths simulate information extraction from horizontal, vertical, and two diagonal directions, allowing VMamba to efficiently capture spatial relationships and global contextual information in the image. During the processing in the SS2D module, the data on each scanning path undergoes an independent Selective State Space transformation and is finally recombined through a Cross-Merge operation to form the final two-dimensional feature map. This innovative design allows VMamba to efficiently integrate information from different directions while maintaining linear computational complexity, making it better suited for various visual tasks.

**Figure:**
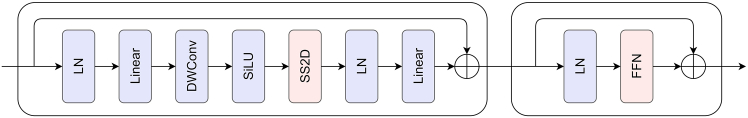
VSS block

**Figure:**
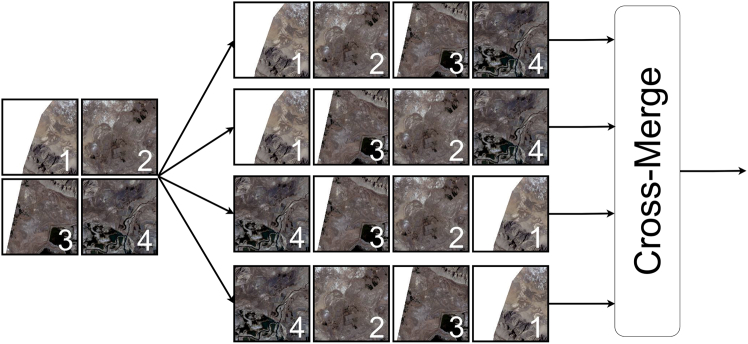
SS2D mechanism

Our encoder uses a siamese architecture, consisting of two branches with shared weights. The two branches have identical structures and are both composed of the VMamba model. The input data of each branch undergoes processing in four stages to progressively extract multi-level feature information. In each stage, the input data first undergoes a downsampling operation to reduce the computational load and improve computational efficiency. Then, the data enters several VSS modules to capture and integrate global information at different levels. Throughout the process, after each stage, the height and width of the feature map are halved, while the number of channels is doubled. Finally, after four stages, each branch obtains four feature maps with global information for subsequent tasks.

#### Difference module

We use four difference modules to capture the differences between the features at different levels output by the encoder. The difference module is shown in Figure 10. Features at different scales can be regarded as mappings of the input sequence within a multi-resolution space, and the discrepancies between these scales reflect the responses of spatiotemporal changes at different frequency levels. By modeling the differences across multi-scale features, the module is capable of simulating the dynamics of temporal variations at both local and global levels, thereby capturing cross-scale spatiotemporal dependencies more effectively. Moreover, the differencing operation can be interpreted as an explicit estimation of spatiotemporal gradients, enabling the model to learn optimal representations of change in an end-to-end training manner. The equation is as follows:

(Equation 6) $$\begin{array}{c} F_{diff}^{i}=ReLu\left(BN\left(Conv2D\left(Concat\left(F_{T_{1}}^{i},F_{T_{2}}^{i}\right)\right)\right)\right) \end{array}$$

where *Concat* represents the concatenation operation.

**Figure:**
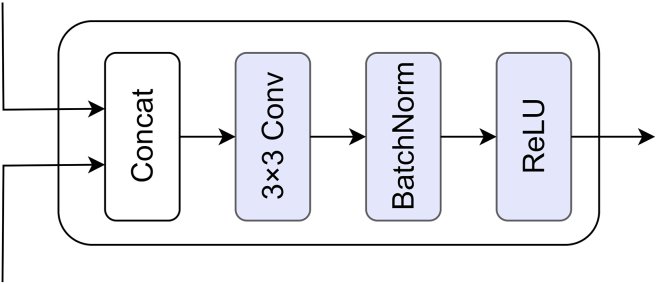
Difference module

#### Decoder

We utilize a decoder that combines MLP and MSSC module (Figure 11) to fuse multi-level difference features and predict the change detection results. First, an MLP layer is used to unify the channel dimensions of the multi-level difference feature maps, followed by upsampling each difference feature map to a size of *H/4 × W/4*. Then, the upsampled difference feature maps are fused, and the dimensions are mapped from *H/4 × W/4 × 4C*_*ou*t_ to *H/4 × W/4 × C*_*out*_ using an MLP layer, reducing channel redundancy and enhancing cross-dimension feature compatibility. Next, the fused feature maps are further processed through the MSSC module for multi-scale feature extraction and key feature enhancement, which strengthens the ability to extract features of small change regions. The output of the MSSC module is then further upsampled to the size of *H × W* to match the resolution of the original input image. Finally, a classifier predicts the change mask map with a resolution of *H × W × N*_*class*_, yielding the final change detection result.

**Figure:**
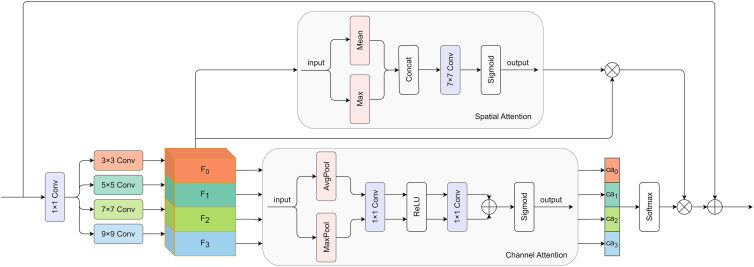
MSSC module

The core of the decoder is the MSSC module. The input is first passed through a 1 *×* 1 convolution to adjust the channel dimensions, and then processed by four parallel convolutional branches for multi-scale feature extraction. The kernel sizes of each branch increase according to *k*_*i*_ *=* 2 *×* (*i +* 1) *+* 1 (where *i* = 0, 1, 2, 3), and a grouped convolution strategy is employed to dynamically adjust the number of groups *G*_*i*_ = 2 *×* (*k*_*i*_ - 1) / 2 to reduce the amount of calculation and the number of parameters. Next, the Channel Attention (CA) module selectively weights the importance of each channel, thereby optimizing feature representation. Then, the weights of the multi-branch are normalized through Softmax to generate the cross-scale dynamic allocation weight *W*. Additionally, the multi-scale features from the four branches are concatenated, and the Spatial Attention (SA) module is used to learn the important regions in space, enhancing the feature representation of key areas. Then, the concatenated multi-scale features *F* are element-wise multiplied with the output *sa* from the SA module, followed by an element-wise product operation with the weights *W*. Finally, the resulting product is added element-wise to the initial input of the MSSC module. The equation is as follows:

(Equation 7) $$\begin{array}{c} F_{i}={\;Conv}_{i}\left(k_{i}\times k_{i},G_{i}\right)\left({Conv}_{1\times 1}\left(X\right)\right)\;i=0,1,2,3 \end{array}$$

(Equation 8) $$\begin{array}{c} {ca}_{i}=CA\left(F_{i}\right)\; \end{array}$$

(Equation 9) $$\begin{array}{c} W=Softmax\left(Concat\left({ca}_{i}\right)\right) \end{array}$$

(Equation 10) $$\begin{array}{c} sa=SA\left(Concat\left(F_{i}\right)\right) \end{array}$$

(Equation 11) $$\begin{array}{c} out=sa⊙F⊙W+X \end{array}$$

where *X* is the input of the MSSC module, *F* represents the multi-scale feature map, the size of the *i*-th convolutional kernel is *k*_*i*_ *=* 2 *×* (*i +* 1) *+* 1 and *i*-th group size *G*_*i*_ = 2 *×* (*k*_*i*_ - 1) / 2, *W* denotes the channel attention weights for the feature map *F*, ca and sa are the outputs of the CA and SA modules respectively, ⨀ represents the Hadamard product.

Channel attention focuses on the channel dimension of the feature map. By learning the relationships among channels, the weights of significant channels are enhanced while those of less relevant channels are suppressed, thereby optimizing the feature representation. The input feature map undergoes global average pooling and global max pooling to encode global spatial information and capture significant feature cues. These pooled features are then processed by a shared network composed of convolutional layers and a ReLU activation function. Finally, the two processed features are element-wise added and passed through a Sigmoid activation function to generate the channel attention map. The formula is as follows:

(Equation 12) $$\begin{array}{c} CA\left(F\right)=Sigmoid\left(FC\left(AvgPool\left(F\right)\right)+FC\left(MaxPool\left(F\right)\right)\right) \end{array}$$

Spatial attention emphasizes the spatial dimension of the feature map. By learning the important regions in the space, the feature representation of the key regions is enhanced. The input feature map first applies average pooling and maximum pooling along the channel dimension to extract features. After concatenating the two resulting feature maps, a 7×7 convolution operation is applied to extract the spatial relationship. Finally, the spatial attention map is generated via the Sigmoid activation function. The formula is as follows:

(Equation 13) $$\begin{array}{c} SA\left(F\right)=Sigmoid\left(Conv7\times 7\left(Concat\left(Mean\left(F\right),\mathrm{Max}\left(F\right)\right)\right)\right) \end{array}$$

### Quantification and statistical analysis

The deep learning networks were implemented using Python 3.10 and PyTorch 2.1.0. All computations were performed within the Python programming environment. The graphical abstract and other figures presented in this study were generated using Python-based libraries and Draw.io.
